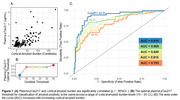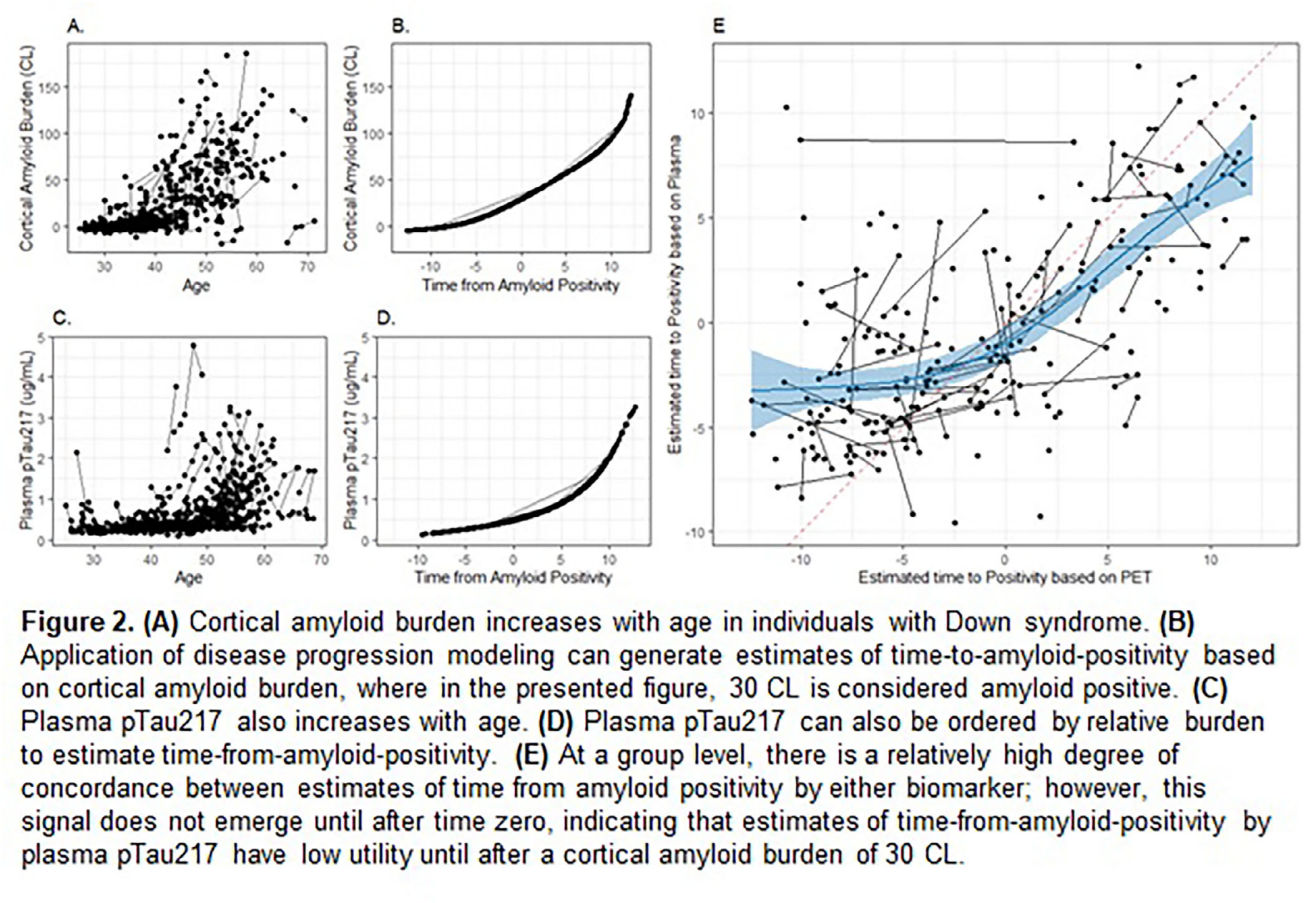# Plasma pTau217 as a marker of both the current and future state of amyloid pathology in adults with DS

**DOI:** 10.1002/alz70856_099356

**Published:** 2025-12-25

**Authors:** Julie K. Wisch, Ziqiao Jiao, Patrick J. Lao, Natalie C. Edwards, Adam Brickman, Ann D Cohen, Zinayida Schlachetzki, Melissa Petersen, Benjamin L Handen, Bradley T Christian, Mark Mapstone, H. Diana Rosas, Florence Lai, Joseph H. Lee, Sharon J. Krinsky‐McHale, Frederick A Schmitt, Jordan P. Harp, Christy L. Hom, Ira T. Lott, Sigan L Hartley, Shahid Zaman, Lauren Ptomey, Jeffrey M. Burns, Laura Ibanez, Michael S. Rafii, Elizabeth Head, Beau Ances, The Alzheimer's Biomarker Consortium ‐ Down Syndrome

**Affiliations:** ^1^ Washington University in St. Louis School of Medicine, St. Louis, MO, USA; ^2^ Washington University, St. Louis, MO, USA; ^3^ Columbia University Irving Medical Center, New York, NY, USA; ^4^ Columbia University, New York, NY, USA; ^5^ University of Pittsburgh School of Medicine, Pittsburgh, PA, USA; ^6^ University of Southern California, Los Angeles, CA, USA; ^7^ Institute for Translational Research, University of North Texas Health Science Center, Fort Worth, TX, USA; ^8^ University of Pittsburgh, Pittsburgh, PA, USA; ^9^ University of Wisconsin‐Madison, Madison, WI, USA; ^10^ University of California, Irvine, Irvine, CA, USA; ^11^ Massachusetts General Hospital, Harvard Medical School, Boston, MA, USA; ^12^ Harvard/Massachusetts General Hospital, Boston, MA, USA; ^13^ Columbia University Medical Center, New York, NY, USA; ^14^ New York State Institute for Basic Research in Developmental Disabilities, Staten Island, NY, USA; ^15^ University of Kentucky College of Medicine Department of Neurology, Lexington, KY, USA; ^16^ University of Kentucky / Sanders‐Brown Center on Aging, Lexington, KY, USA; ^17^ Waisman Center, University of Wisconsin‐Madison, Madison, WI, USA; ^18^ University of Cambridge, Cambridge, United Kingdom; ^19^ University of Kansas Medical Center, Kansas City, KS, USA; ^20^ University of Kansas Alzheimer's Disease Research Center, Kansas City, KS, USA; ^21^ Washington University in St. Louis, School of Medicine, St. Louis, MO, USA; ^22^ Alzheimer's Therapeutic Research Institute, Keck School of Medicine, University of Southern California, San Diego, CA, USA

## Abstract

**Background:**

Potential anti‐amyloid therapy trials for Down syndrome (DS) require PET‐confirmed “amyloid positivity” for inclusion. Disease progression modeling predicts when individuals with low but meaningful amyloid levels will meet enrollment criteria. Predictions of time‐to‐biomarker‐threshold rely on published differential equation techniques. Time‐to‐amyloid‐positivity estimates have been published using longitudinal amyloid PET data. Although plasma pTau217 effectively determines amyloid plaque presence dichotomously, its ability to replace PET measures for forecasting future trial eligibility is unknown. Sensitivity at low amyloid levels is critical, as trials target early intervention.

**Method:**

We included 329 people with DS enrolled in ABC‐DS, 167 completed one or more amyloid PET (PiB or AV45) and plasma pTau217 (Lilly assay) evaluations. The remaining participants completed either amyloid PET (*N* = 99) or venipuncture (*N* = 63). The optimal plasma pTau217 value for detecting amyloid positivity was evaluated at various thresholds using ROC analysis, maximizing Youden Index. Disease progression modeling estimated time‐to‐amyloid‐positivity using longitudinal amyloid PET and plasma pTau217 data. Temporal estimates for the two methods were compared using generalized additive models. We compared predictions of time‐to‐amyloid‐positivity using Mean Average Error (MAE) and Spearman correlations.

**Result:**

A plasma pTau217 threshold of 0.4778 ug/mL was the optimal cutoff for amyloid positivity across 15 – 30 Centiloids (CL). Although 12 CL was the best‐performing cutoff (0.2271 µg/mL), its apparent high AUC is misleading, as the dataset imbalance (152 amyloid‐positive cases) results in 109 false positives out of 167 samples. Excluding 12 CL, plasma pTau217 accuracy improved with increasing cortical amyloid burden (Figure 1). At lower cortical amyloid levels (< 30 CL), plasma pTau217 was highly variable (MAE = 5.2 years, ρ = 0.226). Beyond 30 CL, plasma and PET estimates of time‐to‐positivity correlated well (MAE = 3.5 years; ρ = 0.629) (Figure 2).

**Conclusion:**

Plasma pTau217 effectively predicts amyloid‐PET positivity in individuals with DS and generates continuous estimates of time‐to‐amyloid‐positivity in individuals with cortical amyloid burden > 30 CL. However, it is not as useful for estimating time‐to‐positivity at lower amyloid pathology levels (10 – 30 CL). This specific plasma pTau217 assay may lack sensitivity for detecting early amyloid positivity in people with DS.